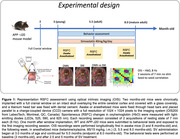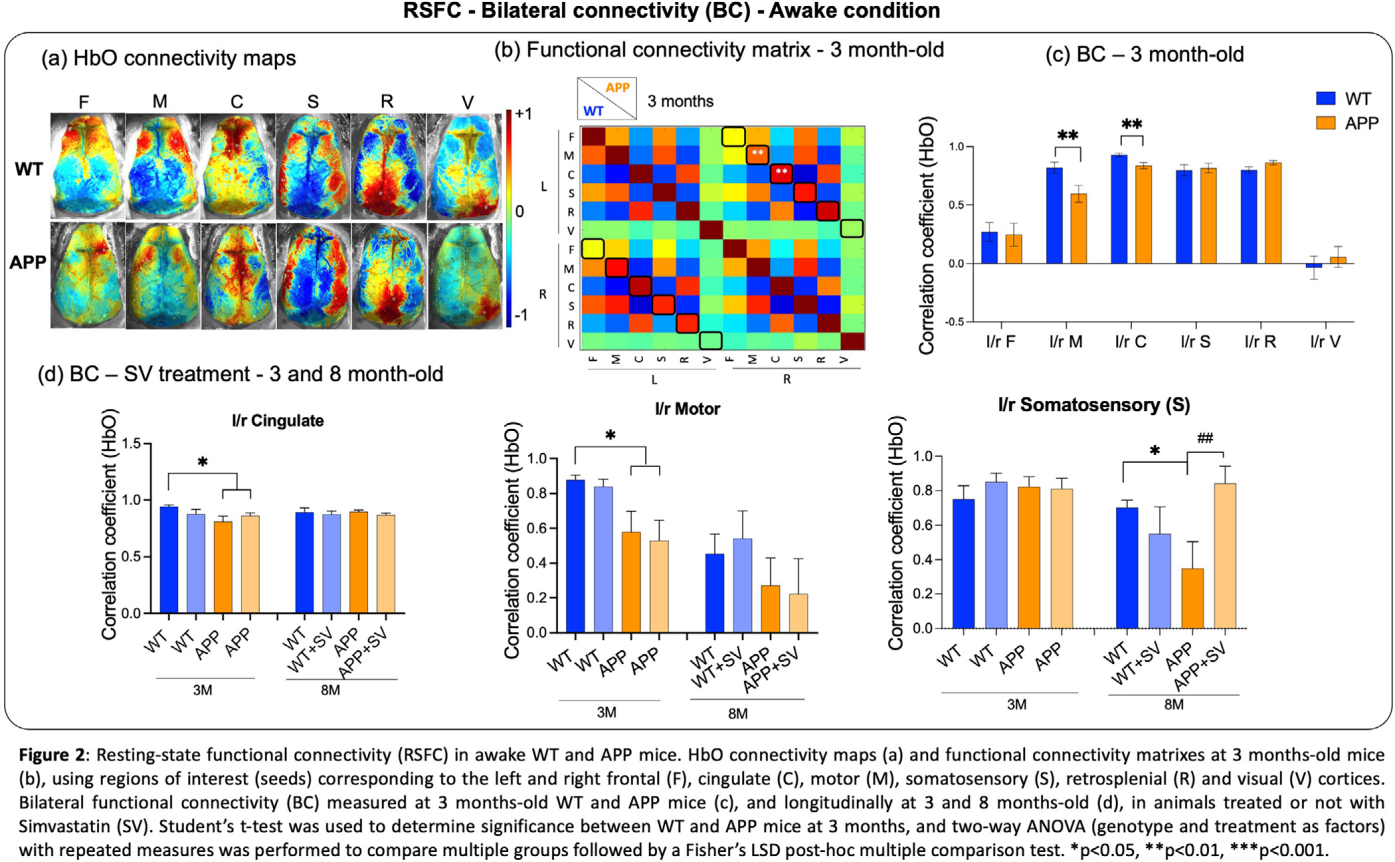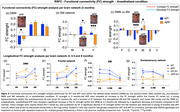# Functional connectivity changes through Alzheimer's disease continuum: disease onset, progression, and response to therapy

**DOI:** 10.1002/alz.094088

**Published:** 2025-01-09

**Authors:** Aline R. Zimmer, Miled Bourourou, Frédéric Lesage, Edith Hamel

**Affiliations:** ^1^ Federal University of Rio Grande do Sul, Porto Alegre, Rio Grande do Sul Brazil; ^2^ McGill University, Montreal, QC Canada; ^3^ École Polytechnique de Montréal, Montréal, QC Canada; ^4^ Montreal Neurological Institute, Montreal, QC Canada

## Abstract

**Background:**

Hemodynamic signals are the basis of functional brain imaging techniques, such as fMRI and NIRS, and are often used to infer changes in resting‐state functional connectivity (RSFC) in Alzheimer’s disease (AD) and other dementias. Increasing evidence suggests that disruption of neuronal circuits has been associated with the AD continuum and may precede changes in Ab and tau biomarkers, neurodegeneration, and cognitive impairment. To better understand the changes in brain RSFC through the AD spectrum, we use hemodynamic signals to detect disease onset, progression, and response to therapy in a mouse model of AD.

**Method:**

Hemodynamic signals were recorded longitudinally (3‐8 months) in anesthetized (ketamine/xylazine) and awake WT and APP (J20) mice, implanted with full cranial window, using optical imaging of intrinsic signals (OIS), together with cognitive testing. Seed‐based functional connectivity maps were generated for the bilateral connectivity (BC) and RSFC analyses. The effects of simvastatin (SV), a cardiovascular medicine that has shown promise in preventing dementia, were evaluated after 2.5 and 5 months.

**Result:**

Alterations in RSFC in brain regions associated with the sensory‐motor (SM) and default‐mode (DMN) networks were detected before the appearance of cognitive impairment. 3‐month‐old APP mice showed consistent decrease in bilateral functional connectivity (BC) in motor (M) and cingulate (C) cortical regions and a severe hypoconnectivity within the SM network in the RSFC analysis. Throughout the course of the disease, RSFC analysis in APP mice uncovered an early hyperconnectivity within the DMN, mainly driven by the frontal (F) cortex, followed by a later hypoconnectivity stage. At late stage of the disease (8‐months‐old), a decreased BC in somatosensory (S) cortex was detected. SV treatment prevented the aberrant increases in DMN FC in midlife APP mice, improved the BC of S cortex, while concurrently sparing cognitive function.

**Conclusion:**

Our results demonstrated that hemodynamic signals measured by OIS at the cortical level successfully detected RSFC disruptions preceding dementia in APP mice and allowed to capture SV therapeutic benefits. These findings suggest that OIS, or its human equivalent NIRS, could contribute to effectively diagnosing the early stages of AD, leading to promising intervention opportunities.